# Therapy Effects of Bone Marrow Stromal Cells on Ischemic Stroke

**DOI:** 10.1155/2016/7682960

**Published:** 2016-03-16

**Authors:** Xinchun Ye, Jinxia Hu, Guiyun Cui

**Affiliations:** Department of Neurology, The Affiliated Hospital of Xuzhou Medical College, Xuzhou, Jiangsu Province 221006, China

## Abstract

Stroke is the second most common cause of death and major cause of disability worldwide. Recently, bone marrow stromal cells (BMSCs) have been shown to improve functional outcome after stroke. In this review, we will focus on the protective effects of BMSCs on ischemic brain and the relative molecular mechanisms underlying the protective effects of BMSCs on stroke.

## 1. Introduction

Stroke is the second most common cause of death and major cause of disability worldwide [1]. Current therapies of stroke target angiogenesis, neurogenesis, and oligodendrogenesis to attenuate brain tissue damage from ischemic injury and improve neurological outcome. However, the only globally approved drug for acute ischemic stroke is tissue plasminogen activator (tPA), but it must be given within 4.5 h after stroke onset [2]. Thus, the beneficial effect of tPA is limited to a narrow therapeutic window.

Recently, many other agents have provided promising approaches for stroke patients beyond the hyperacute phase in clinical trials. Statins used to lower circulating cholesterol levels have been shown to possess neurorestorative properties, increase angiogenesis and neurogenesis after stroke, and improve functional recovery [3]. Niaspan, primarily used to provide vitamin B3, has also been shown to reduce blood–brain barrier (BBB) leakage and improve neurological outcome and vascular remodeling after stroke [4, 5].

Cell-based therapy is a promising approach to promote functional recovery after stroke [6]. Bone marrow stromal cells (BMSCs) treatment of stroke has been shown to enhance function recovery, ameliorate cognitive dysfunction, and improve neuroplasticity by regulating neurogenesis, angiogenesis, and oligodendrogenesis [7–12].

This review will focus on the characteristics of BMSCs treatment for stroke and molecular mechanisms of neurorestorative effects of BMSCs on ischemic stroke.

## 2. Identification and Characteristics of Bone Marrow Stromal Cells

Stem cells were firstly isolated from bone marrow (BM) in 1960s, characterized by both their rapid adherence to plastic and their fibroblast-like morphology [13]. It was examined in the laboratory that the BMSCs contain many subsets, including hematopoietic stem cells (HSCs), mesenchymal stem cells (MSCs), endothelial progenitor cells (EPCs), and very small embryonic-like stem cells (VSELs) [14–16]. BMSCs express a number of nonspecific markers as CD105 (SH2), CD73 (SH3/4), CD44, CD90 (Thy-1), CD71, and Stro-1, as well as the adhesion molecules CD106 and CD166, intercellular adhesion molecule1, and CD29 [17].

BMSCs are characterized with paracrine actions, immunomodulation, and multipotency [18–20]. The neurotrophic effects of BMSCs, releasing growth and trophic factors or stimulating their release from resident brain cells, have been suggested to be beneficial in ischemic stroke [21]. The neurotrophic factors, including hepatocyte growth factor (HGF), vascular endothelial growth factor (VEGF), brain-derived neurotrophic factor (BDNF), basic fibroblast growth factor (bFGF), and insulin growth factor-1 (IGF-1), exert several protective effects, such as angiogenesis, neurogenesis, neuroprotection, and synaptogenesis [19].

BMSCs have the potential to be differentiated into mesenchymal lineages, neurons, and glial cells [22]. They can express neural or glial protein markers and take place of dying cells in the ischemic brain after treatment of stroke with BMSCs in animal experiments [23]. Some studies demonstrate that transplanted BMSCs can also differentiate into vascular endothelial cells in ischemic brain [24, 25].

The immunomodulatory function is also considered to account for the beneficial effects of BMSCs. BMSCs have a profound inhibitory effect on T-cell proliferation of inflammatory state, which modulate an immune response by inhibiting antigen-specific T-cell proliferation and cytotoxicity [26]. The underlying mechanisms of immunosuppressive effect of BMSCs involve several soluble molecules such as nitric oxide, indoleamine 2,3-dioxygenase, transforming growth factor-*β*1 (TGF-*β*1), HGF, interleukin- (IL-) 10, IL-6, and soluble HlA-G5, which are partially understood [27–29].

## 3. Ischemic Stroke

In the acute phase after stroke, ischemic injury is a following step through BBB breakdown, neuronal damage, and astrocytes activation [30–32]. Increased BBB permeability starts 2 h after the onset of ischemia; the dissolution of endothelial basal lamina may result from accumulation of vascular endothelial growth factor, active matrix metalloproteinases, and other protease activities following neurons, glia, and endothelial cells injury [33–36]. The second phase of severe BBB injury occurs within 24–72 h after ischemic stroke [37]. This stage is characterized with leukocyte infiltration and danger-associated molecular patterns (DAMPs) released from the necrotic brain to activate infiltrating immune cells [38].

High mobility group box 1 (HMGB1), a kind of DAMPs, is localized in cell nuclei in the normal brain. It can translocate into the cytosol and extracellular space after ischemic stroke [39]. Release of HMGB1 is observed 2–4 h after stroke onset and peaks around 4 days by activated microglia and astrocytes [40, 41]. Extracellular HMGB1 can bind to its receptors and activate downstream proinflammatory molecules such as TNF-*α*, IL-1*β*, MMP9, and RAGE [42–46]. The increased expression of these proinflammatory molecules contributes to BBB disruption, facilitates immune cell migration, and forms signaling complexes in the ischemic brain [47]. Blockade of HMGB1 mediated inflammatory signaling during the acute phase of stroke is beneficial for BBB functional integrity and functional recovery [21, 46, 48].

## 4. The Effects of BMSCs on Cerebral Ischemia

Damaged brain can be surprisingly plastic and crosstalk between various types of remodeling in ischemic brain occurs after stroke [49]. Angiogenesis is defined as the formation of new capillaries from preexisting vessels through the activation of hypoxia inducible factor-1*α* and proangiogenic molecules such as vascular endothelial growth factor-A (VEGF-A) and VEGF receptor-2 (VEGFR-2), angiopoietins (Ang-1 and Ang-2), cognate receptor Tie-2, neuropilin-1, and basic fibroblast growth factor [21, 50–52]. Ang-1 plays a vital role in recruiting pericytes and basement membrane deposition to inhibit endothelial cell migration, keep vascular stability, and form tube-like structures. However, Ang-2 acts as an antagonist for Ang-1 and balances the neovascularization procession [51]. Our previous study indicated that pro- and antiangiogenic factors play a vital role in the procession of angiogenesis after ischemic stroke. Enhanced secretion of angiogenic cytokines after treatment with BMSCs in ischemic stroke has strong angiogenic effects on microvasculature remodeling in neovascularization [53].

In the past decade, it was claimed that BMSCs treatment not only exerts angiogenic effects, but also induces neurogenesis, axonal sprouting, and neurite outgrowth after stroke [54]. Several possible mechanisms, involved in BMSCs-induced neuroprotective effects, are as follows. On the one hand, BMSCs treatment can induce the generation of new neurons from progenitor cells within the subventricular zone (SVZ) of the lateral ventricle and the dentate gyrus in ischemic brain [22, 55]. On the other hand, BMSCs can enhance neurogenesis, oligodendrogenesis, and synaptogenesis by differentiating into neurons and oligodendrocyte [56].

## 5. BMSCs Treatment of Ischemic Stroke in Aged Animals

Age is the principal nonmodifiable risk factor for stroke [57]. Studies have shown that the rates of functional impairment and mortality were significantly increased in aged stroke patients when compared to relative young ones [58–62]. Studies have shown that the proliferation of endogenous neural precursor cells (NPCs) was significantly increased in the SVZ after BMSCs treatment in aged stroke animals [63, 64]. Recently, BMSCs treatment of stroke has also shown to ameliorate neurological impairment in aged stroke rats by reducing infarction volume and promoting angiogenesis, neurogenesis, and synaptogenesis [65].

## 6. BMSCs Treatment in Diabetic Stroke

Diabetes mellitus (DM), as a global health problem complicated with microvascular and macrovascular diseases, is a predisposing risk factor for stroke. Stroke in diabetic patients has a higher mortality and worse outcomes after stroke [66–68]. Previous study indicates that type 1 diabetic rats exhibited increased mortality and BBB leakage and reduced functional recovery when compared to nondiabetic individuals [69]. The poor outcomes after stroke in type 1 diabetic rats have been attributed to HMGB1 mediated inflammatory response and Ang1 regulated angiogenesis [70, 71].

BMSCs treatment of stroke has also been reported to contribute to increased dysfunctional angiogenesis and the risk of cerebral hemorrhage after stroke in type 1 diabetic rats [72–75]. This dysfunctional angiogenesis was associated with reduced functional recovery and vessel wall maturity, increased mortality rate, BBB leakage, and brain hemorrhage after diabetic stroke [11, 76].

## 7. Administration Strategies of BMSCs

Behavioral improvements after treatments of stroke with BMSCs have been observed with intracerebral, intracerebroventricular, and intravascular deliveries of stem/progenitor cells. Here we discuss the pros and cons of different delivery strategies.

Intracerebral and intracerebroventricular injections result in more transplanted cells in the infarcted region when compared to other delivery routes. However, the procedural risk for injection significantly raises safety issues [77, 78]. Until now, intracerebroventricular injection has been used only in one clinical trial. The study showed some patients developed fever and meningeal signs after cell implant via intracerebroventricular delivery [79]. Intravascular injection is another strategy to deliver BMSCs in stroke animals and patients. This strategy has several advantages, including easy injection and potential for widespread BMSCs distribution [80]. However, intravascular routes also have safety problems. BMSCs may stick together and cause microemboli, including lethal pulmonary emboli and microstrokes [80].

In conclusion, each cell delivery method has its pros and cons. Stroke subtype and cell delivery timing and working mechanisms should be taken into consideration together with the selection of cell delivery route.

## 8. Therapeutic Time Window

The optimal time for BMSCs delivery may be dependent on their mechanism of action. If a treatment focuses on neuroprotective mechanisms, acute delivery will be very important. If BMSCs aim to enhance endogenous repair mechanisms, then subacute transplantation would be optimum as these events are more prevalent in the first few weeks after stroke [81]. The route of administration may also affect the timing of transplantation. Because inflammatory signals may guide BMSCs home to the ischemic brain, intravascular injection may require early administration [71, 82]. In conclusion, how the timing of administration affected the outcome of these trials is not clear, but they at least demonstrate that delivery of cells at different times is feasible.

## 9. Conclusion

Taken together, although tPA is the only approved treatment for acute ischemic stroke, cell-based therapy, especially BMSCs-based therapy, has also been shown to enhance function recovery, ameliorate cognitive dysfunction, and improve neuroplasticity after stroke. Many factors including nonmodifiable and modifiable risk factors such as age and diabetes may affect the efficiency of BMSCs treatment of stroke. Further investigations into the use of BMSCs as a therapeutic agent for the treatment of stroke are warranted.
